# Hospitals' Reply to Lord Crawford

**Published:** 1919-08-30

**Authors:** W. Webster Smith

**Affiliations:** Secretary. Richmond, Whitworth, and Hardwicke Hospitals, Dublin.


### DUBLIN HOSPITALS AND THE GOVERNMENT.

### Hospitals' Reply to Lord Crawford.

THE Richmond, Whitworth, and Hardwicke, known as the House of Industry Hospitals, Dublin, which have decided to close because they cannot fall further into debt, have appealed in vain, as THE HOSPITAL has recorded, for an increase of the Government grant. The matter was raised in the House of Lords, and after the Earl of Crawford's reply for the Government was published, Mr. W. Webster Smith, secretary, was instructed to issue the following statement on behalf of the joint hospital board to the Press :

As the answers, as reported in the Press, which Lord Shandon received from the Earl of Crawford in the House of Lords, to the questions concerning the financial difficulties of these hospitals, are very misleading, I have been asked to send you the following explanatory note :

The Earl of Crawford implies that the House of Industry Hospital grant dates only from 1856. The fact is that the Parliamentary grant to the hospitals dates from thirty years before the Union; that the amount of same varied with the necessities of the hospitals from 1800 to 1856—the average annual grant for the five years preceding 1856 being £11,719 12s.—and that it was only in 1856 that the grant was reduced to £7,600, a sum supposed at that time to be sufficient, but evidently insufficient now. By the Act of 1856 the government of the hospitals was retained in the hands of the Lord Lieutenant, who alone could nominate Governors. The Treasury Pension Regulations were applied to all permanent salaried offices in the hospitals, and every foot of ground and every stone in the hospitals was vested in the Board of Works. These conditions would appear to render the hospitals a State institution, and certainly militate against their recognition by the public as on the same plane as the ordinary charitable hospitals.

With regard to the extra amounts of £7,000 and £6,000 referred to by Lord Crawford, these sums were paid, as they were to all similar institutions, for the maintenance of sick and wounded soldiers—a matter of loss rather than profit to the hospitals, seeing that last year it cost £119 1s. 10d. *per* occupied bed *per annum*, as against the sum received from the military of £86 13s. 9d.

Finally, Lord Crawford is reported to have said that the hospitals "received an income from endowments amounting to £150,000." There is not a shadow of foundation for this statement. The only permanent endowment outside the Parliamentary grant which the hospitals possess is a sum invested in Consols which brings in almost exactly one hundred pounds a year—and which by the terms of the original bequest cannot be realised.

May I add, to avoid any risk of imposture through the country, that no one has been authorised to collect money for the hospitals? This is not to say that we shall the less esteem the assistance of any kind friends who will help to tide us over the present period of difficulty.

W. WEBSTER SMITH, Secretary.
Richmond, Whitworth, and Hardwicke Hospitals,
Dublin.

